# Plug-and-Play Lymph Node-on-Chip: Secondary Tumor Modeling by the Combination of Cell Spheroid, Collagen Sponge and T-Cells

**DOI:** 10.3390/ijms24043183

**Published:** 2023-02-06

**Authors:** Sergei V. German, Anatolii A. Abalymov, Maxim A. Kurochkin, Yuliya Kan, Dmitry A. Gorin, Marina V. Novoselova

**Affiliations:** 1 Skolkovo Institute of Science and Technology, Bolshoy Boulevard 30, bld. 1, 121205 Moscow, Russia; 2 Institute of Spectroscopy of the Russian Academy of Sciences, Fizicheskaya, 5, 108840 Moscow, Russia; 3 Science Medical Center, Saratov State University, Astrakhanskaya 83, 410012 Saratov, Russia

**Keywords:** organ-on-chip, cell spheroid, microfluidic, lymph node

## Abstract

Towards the improvement of the efficient study of drugs and contrast agents, the 3D microfluidic platforms are currently being actively developed for testing these substances and particles in vitro. Here, we have elaborated a microfluidic lymph node-on-chip (LNOC) as a tissue engineered model of a secondary tumor in lymph node (LN) formed due to the metastasis process. The developed chip has a collagen sponge with a 3D spheroid of 4T1 cells located inside, simulating secondary tumor in the lymphoid tissue. This collagen sponge has a morphology and porosity comparable to that of a native human LN. To demonstrate the suitability of the obtained chip for pharmacological applications, we used it to evaluate the effect of contrast agent/drug carrier size, on the penetration and accumulation of particles in 3D spheroids modeling secondary tumor. For this, the 0.3, 0.5 and 4 μm bovine serum albumin (BSA)/tannic acid (TA) capsules were mixed with lymphocytes and pumped through the developed chip. The capsule penetration was examined by scanning with fluorescence microscopy followed by quantitative image analysis. The results show that capsules with a size of 0.3 μm passed more easily to the tumor spheroid and penetrated inside. We hope that the device will represent a reliable alternative to in vivo early secondary tumor models and decrease the amount of in vivo experiments in the frame of preclinical study.

## 1. Introduction

A conservative biology implies operating with two-dimensional (2D) cell cultures, which obviously has a low correlation with real three-dimensional (3D) biological tissues, because 2D cell colonies poorly represent the specific microenvironment of 3D living systems [1,2], does not take into account spatial cellular interactions [3] and ignores hydrodynamic features of real biological tissues [1,2]. This factor becomes critical when working with cellular models of cancer metastases, which leads to an incorrect assessment of the drug effectiveness, and accordingly, to a high percentage of rejected drugs already at the next stages [1]. The number of unknown factors in the transition from 2D cultures to laboratory animals increases dramatically, which leads to a situation where an overall failure rate in drug development exceeds 96%, including 90% failure during clinical development [4]. Thus, it has been estimated that only 1 in 10,000 new chemical compounds receive FDA approval and are brought to market [5,6]. Thus, discrepancies between in vitro and in vivo scientific data and clinical conditions suggest that new models should reproduce human pathophysiological processes.

In the tissue engineering (TE) paradigm, the tools of engineering and life sciences come together to develop bioartificial organ and tissue substitutes, which, in turn, can be used in regenerative medicine, pharmaceuticals, diagnostics and basic research to elucidate fundamental aspects of cellular function [7]. The design of 3D tissue models is currently under development, demonstrating high potential in overcoming the limitations of already available models [8]. Nevertheless, many questions still remain open regarding determining the optimal materials for framework formation, cell source and bioproduction technology, as well as the best cell culture conditions for accurate reproduction of native tissue and the environment [9]. In the near future, three-dimensional tissue engineering models are expected to become useful tools for pre-testing and screening drugs and treatments, as well as for investigating the molecular mechanisms underlying disease onset and progression. At this point, tissue-engineered constructs can be developed both through the use of biomaterials [10], external influences such as magnetic fields [11] and bioactive molecules that would accelerate cell integration processes into native tissues [12,13]. In tissue engineering constructs, cells can be in the biomaterial in different forms: single [14], monolayers [15] and spheroids [16], which depends on the purpose of the study.

The combination of the aforementioned limitations of the 2D structure has pushed research towards the development of so-called organ-on-a-chip systems—3D microfluidic device that combines micro-manufacturing and tissue engineering to replicate the critical physiological environment and functions of the human organs [17]. Currently, work is underway to create various organ-on-a-chip systems: “heart on a chip” [18], “liver on a chip” [19], “lungs on a chip” [20], “intestine on a chip” [21], etc.

Microfluidic organ-on-a-chip technology has made it possible to study certain diseases, including cancer [22,23,24,25,26,27]. An essential feature of cancer treatment strategy development is the specifics of cancer cell spreading and the morphology [3]. In many cases cancer metastasis spreads through the lymphatic system [28], and the number of lymph nodes, first of all the sentinel lymph node (SLN), involved in the secondary tumor process is the most prognostic factor for the five-year survival rate of patients [29]. In the last decade, methods for accurately identifying the sentinel lymph node (SLN) have attracted much attention from surgeons and researchers in the field of medical diagnostics. Among the various methods used to SLN detection, various carriers as delivery agents to the LN are attracting increasing attention [30,31]. However, existing contrast agents currently do not meet modern requirements for the accuracy of SLN detection, accessibility, minimal invasiveness, etc. Since SLN is directly interesting from the point of view of cancer diagnostics, as well as the treatment of diseases in which the immune system is involved, one of the promising directions is the creation of its model on a chip. However, despite great efforts made to develop models of immune organs in vitro, the complex architecture of the LN is still not fully reproduced in vitro, even though the LN is the organ in which key interactions of the immune system with medicines and which has great potential for revolutionary drug discovery and development [17,32,33].

The design and development of an in vitro LN model has several aspects to be considered and these include: The development of a 3D mesh that could be a model of the LN extracellular matrix (ECM); applying of dynamic flow conditions [5]. The main processes of lymph filtration in the LN occur in the lymphatic sinuses, the cavities which have a three-dimensional network of cords from collagen. Thus, collagen is a suitable material for modeling the ECM in the lymph node. Collagen is a promising material not only in tissue engineering, but also for creating tissue models. Collagen based membranes received recognition in the organ-on-chip application due to the similarity with the natural ECM for such cases as pancreas [34], lung [35], and lymphoid follicles [34]. Thin membrane made of collagen I type was used in an organ-on-a-chip device where the authors implemented the vitrified collagen to mimic the eye basement membrane [36]. However, a 2D membrane does not recreate well the 3D collagen ECM of a lymph node [37]. Another material, a collagen hydrogel, was mentioned in the recent review dedicated to the development of LNOC devices [5]. However, the pores in the hydrogel are significantly smaller than the pores in the ECM of the lymph node [37]. Thus, a collagen membrane or gel does not correlate well with the structure of a native lymph node. Notably, the collagen could be processed to design the required morphology and structure using freeze-casting and crosslinking. A collagen sponge, produced by freeze-casting possesses the high surface porosity supporting the cell affinity and cultivation which is applicable to modelling the 3D environment of organs.

In connection with the above, the need to develop 3D cell technologies intermediate between 2D cell colonies and in vivo models of laboratory animals, which would take into account the specific 3D morphology of secondary tumor and their interaction with specific health tissues, becomes evident.

We introduce a new optimized Plug-and-Play (PnP) lymph node-on-chip that represents a real in vitro alternative to traditional in vivo studies in mice, including complex environmental factors such as lymph flow and the extracellular collagen matrix. The chip is a reusable microfluidic device containing a three-dimensional spheroid that mimics secondary tumor in a collagen-based sponge microwell.

## 2. Results and Discussion

### 2.1. Design and Fabrication of LNOC

**Figure 1:**
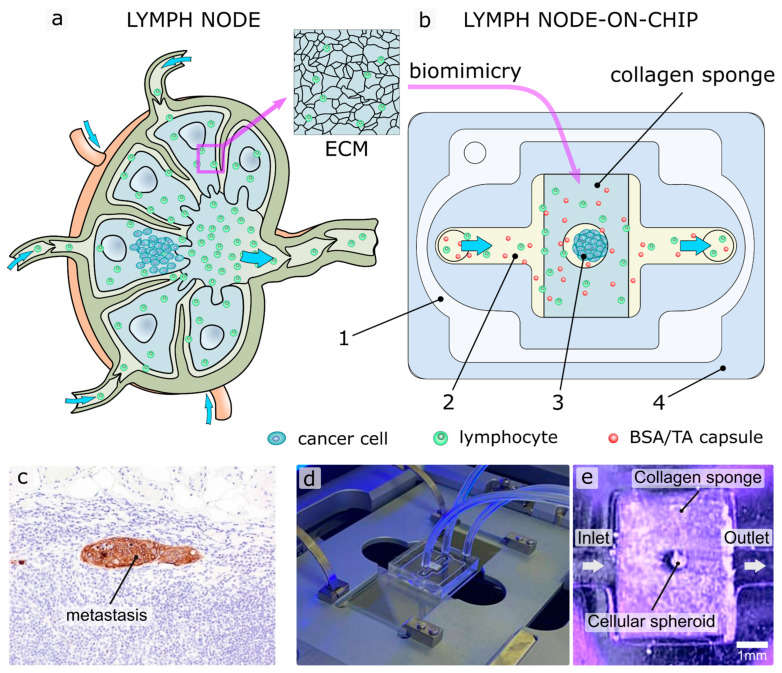
Engineering of LNOC. Schematic of the human native lymph node (**a**) and LNOC structure (**b**), reproducing structural features of LN with secondary tumor. 1—suction cup; 2—main channel; 3—cellular spheroid; 4—chip body. Cells stained in blue—secondary tumor cells, green—lymphocyte; red—capsules. (**c**) Secondary tumor due to metastasis in the sentinel lymph node (keratin stain). Reprinted with permission from Elizabeth Euscher (2020) [38]. (**d**) Photo of chip installed in the Operetta CLM system. (**e**) Macrophotograph of the assembled and ready to use chip.

The main aspect in the development of the chip was the creation of a model of the collagen ECM using a collagen sponge, with a comparable pore size. We suggest the technology of creating microfluidic devices from polydimethylsiloxane elastomer (Supplementary Figure S1), taking into account such functional features of the native LN as a collagen ECM structure and flow of lymphocytes (Figure 1).

On one surface of the chip body, the main channel is formed, through which the culture medium flows (Figure 1b(2)). A growing cell medium is supplied and extracted through fittings from stainless steel and silicon tubes. Moreover, the chip has an additional isolated wide cavity (Figure 1b(1)) in the body around the channel. This cavity is connected to a small vacuum pump and works as a suction cup, pressing the chip body to the glass for microscopy (Figure 1d) while the vacuum pump is working. Thus, the device can be quickly disassembled to replace the collagen sponge and spheroid. Using such plug-and-play construction of the chip, we can place a cellular spheroid (Figure 1b(3)) directly into the hole in the centre of the collagen sponge just before experimenting and removing it after. Thus, we do not need to grow cells inside the chip. In addition, the chip consists of biocompatible materials (PDMS, glass, collagen), which allows for long-term experiments with cells.

Since we use a vacuum suction cup, the device does not have additional clamping elements and is compact, which makes it easy to install the chip on a standard slide holder of a microscope stage or in an incubator system for microscopy to conduct long-term experiments in optimal conditions for cells (Figure 1d). Pre-saturated culture medium can be pumped through the chip to maintain the carbon dioxide level.

In the center of the chip, the main channel has an extension and a replaceable collagen sponge installed into it (Figure 1; Figure 1e). The interconnected open porosity of the collagen sponge mimics the collagen ECM of the LN. It is known that the ECM in a lymph node is composed of a network of fibers and molecules that provide structural support and help to regulate the movement and behavior of the immune cells (Figure 1a). The pores or openings in the ECM, also known as interstitial spaces, can vary in size and shape depending on the location and the state of the lymph node. In general, the size of the pores in the ECM of a lymph node can range from a few micrometers to several hundred micrometers, depending on the type of ECM fibers and the presence of other ECM components, the state of the lymph node (normal or inflamed) [39,40]. The size of the pores in a sinus of lymph node can vary depending on the location and the state of the lymph node. In general, the size of the pores in a normal lymph node can range from 20–50 μm in diameter. These pores allow lymph fluids to flow through the lymph node, where they can be filtered and analyzed by immune cells [41].

**Figure 2:**
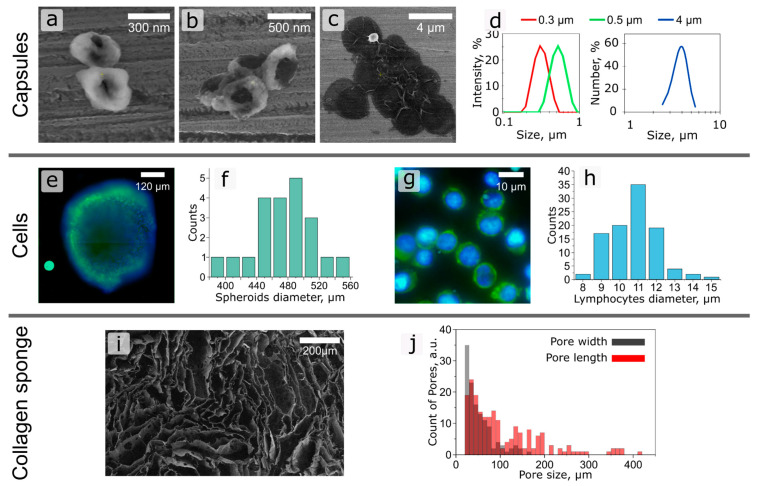
(**a**–**c**) SEM images of polymer capsules of (**a**) 0.3, (**b**) 0.5 and (**c**) 4 µm diameter. (**d**) Plots of size representation of polymer microcapsules obtained by DLS. (**e**) Fluorescence microphotograph of 4T1 cellular spheroid (Alexa Fluor 488 is green fluorescence, Hoechst is blue fluorescence). (**f**) Spheroids size distribution. (**g**) Fluorescence microphotograph of Jurkat cells (Alexa Fluor 488 is green fluorescence, Hoechst is blue fluorescence). (**h**) Jurkat cell size distribution. (**i**) SEM image of collagen sponge. (**j**) Size distribution of meshes in collagen sponge.

Freezing technology was used to obtain a sponge with a suitable pore size [42,43]. By changing the shape of the collagen sponge and the size of its pores, it is possible to more accurately adjust the parameters of the LN model. The SEM of the obtained collagen sponge and the pore size distribution in it are shown in Figure 2i,j. According to the obtained results, the pore sizes fit into the range indicated above.

A model of secondary tumor due to metastasis is recreated by a cellular spheroid placed in the hole in the center of the collagen sponge. To mimic the formation of a secondary tumor in a LN, the 3D human tumor spheroids were chosen, as they most fully reflect the microenvironment of the tumor. Spheroids have a three-dimensional spatial arrangement with enhanced intercellular contact and the ability to form proliferative gradients, hypoxia and necrosis. In this connection, it should be noted that spheroids have three zones: necrotic core, static and proliferative zone. The thickness of the proliferative layer and resting cells can vary from 100 to 220 µm depending on the type of cells [44]. Therefore, it is particularly important to be able to observe how drugs and carriers pass inside the spheroid to the desired depth. Thus, the liquid flows through the channel, and passing through the sponge, interacts with the studied spheroid, which imitates a secondary tumor in the LNs. The complete chip assembly and application process is shown in Video S1.

### 2.2. Study of the Selectivity of Particle Accumulation on LNOC

To assess the functionality of the resulting system, the possibility of using it to assess the penetration of drug delivery systems/contrast agents of various sizes to secondary tumor in the LN was tested. Currently, the use of nanoparticle systems as contrast agents for the detection of SLN, as well as for the delivery of anticancer drugs to tumors, is of great interest, since they have the advantages of selective accumulation in the tumor focus, with the possibility of increasing efficiency and reducing toxicity compared to conventional medical treatment. However, the process of testing these particles is currently being carried out on two-dimensional cell cultures and animals, the disadvantages of which have already been indicated earlier. Thus, we decided to study the size effect of polymer particles based on BSA/TA on the penetration and absorption by tumor cells using the obtained LNOC by perfusion of the obtained particles mixed with lymphocytes through a collagen matrix with a spheroid in the centre. For this, particles (BSA-Cy5/TA)_2_ with a size of 0.3, 0.5, and 4 μm were obtained according to the scheme described above in the “Materials and Methods” section. The SEM and size distribution (DLS) of the obtained capsules are shown in Figure 2a.

The 4T1 breast cancer spheroids used in this study were generated based on the methodology, which was described above in “materials and methods”. As the histogram in Figure 2e shows, the spheroids had an average diameter of 480 µm. Calcein Am (for lymphocytes), Hoechst (for spheroid cell nuclei), and Cy5 (for particles) dyes, which did not interfere with each other’s fluorescence and made it possible to estimate the number and distribution of cells inside the chip, as well as the location of the particles inside a 4T1 spheroid were chosen for cell and capsule imaging. To stain the spheroids, we used Hoechst fluorescent nuclear dye, which can in vivo stain cell nuclei. In the case of studying the immobilization of particles inside spheroids, it is extremely important to work with living cells.

After chip sterilization and spheroid staining, the 4T1 cell spheroid was placed in the center of the chip well (Figure 1e). Next, the construction was installed in the CLM confocal microscope “Operetta” (Perkin Elmer), the fittings were connected through the channels to the syringe pump (Figure 1d). We used T lymphocytes, which are present in the lymph flow, to improve the imitation of the lymph flow. Syringe pump (QHZS-001B, China) was used to insert the mixture of T-cells and capsules (0.3, 0.5 or 4 µm) and fluids into the microfluidic system. The concentration of lymphocytes in the suspension was 1 × 10^6^ cells/mL; the concentration of particles was 1 × 10^9^ particles/mL.

**Figure 3:**
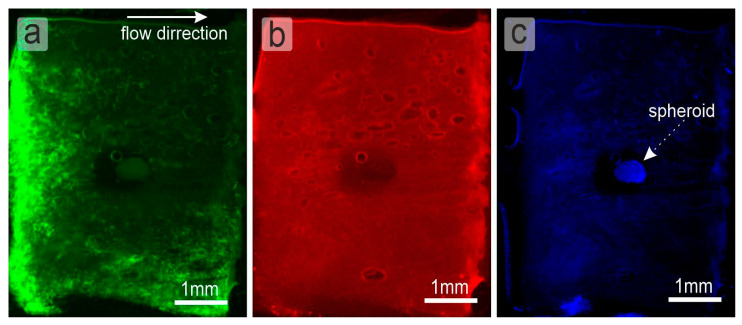
Fluorescence microphotograph of the LNOC during experiment. (**a**) Green fluorescence are Jurkat cells (Calcein AM). (**b**) Red fluorescence are polymer capsules (Cy5). (**c**) Blue fluorescence is 4T1 cellular spheroid (Hoechst). Flow direction is shown in the (**a**) by the white arrow (from **left** to **right**).

The volume flow rate through the LNOC was 0.65 mL/h, assuming that volumetric lymph flow in the rat lymph vessels is in the range of 0.1–1 mL/h [37]. On the individual fluorescence channels, the following became clearly visible: (1) 300 nm capsules evenly filled the collagen sponge and passed through it (Figure 3b). (2) Channels in the sponge are not equal and have some distribution in shape and size, as shown by SEM section of Figure 2i and presented in Figure 2j. However, it can be seen that lymphocytes also pass through the sponge and are present in every part of it. As expected, the maximum fluorescence intensity of lymphocytes is directly opposite the innlet channel of the microfluidic chip (Figure 3a) and gradientally decreases to the borders of the collagen sponge (to the top and to the bottom site).

**Figure 4:**
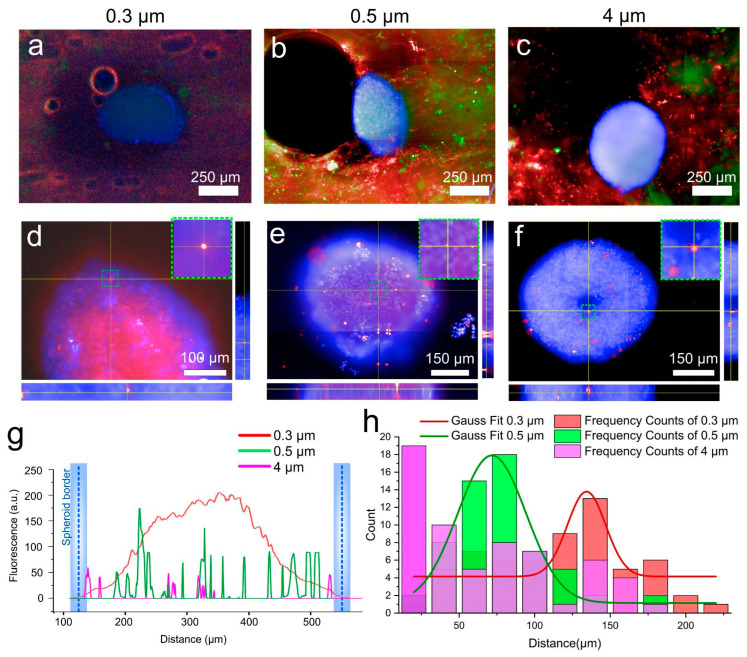
(**a**–**c**) Fluorescence images of chip with 4T1 cell tumor spheroid (Hoechst, blue fluorescence), T lymphocyte cells (Calcein Am, green fluorescence), and (**a**) 0.3, (**b**) 0.5 and (**c**) 4 µm polymer capsules (Cy5, red fluorescence). (**d**–**f**) Orthogonal fluorescence images of cell spheroids with internalized (**d**) 0.3, (**e**) 0.5 and (**f**) 4 µm capsules. (**g**) Photographed fluorescence profiles for cell spheroids with internalized capsules of 0.3, 0.5 and 4 µm. The fluorescence brightness profile along the dashed yellow line in (**d**–**f**), which shows the fluorescence distribution of capsules 0.3 (red), 0.5 (green), and 3.5 (purple) in the cell spheroid, the borders of which are marked with blue. (**h**) The distribution of the capsule’s penetration depth.

The efficiency of capsule passage through the collagen sponge was assessed by confocal microscopy. For this purpose, we first assessed the presence of lymphocyte cells and polymer capsules in the sponge around the cell spheroids (Figure 4a–c). As can be seen from the fluorescence micrographs, capsules and cells successfully pass through the sponge surrounding the spheroid. In all cases, the spheroids slightly change their morphology because they are pressed against the sponge wall by the flow. Such changes in spheroid morphology are considered to be normal in the shear stress for cancer and normal cells in 2D and 3D matrixes [45,46]. The total duration of the experiment was one hour, of which 30 min the spheroid was in the sponge under flow conditions, and 30 min without flow, so that the capsules that hit the membrane could be better absorbed by the surface of the cells. To evaluate the passage of capsules and lymphocyte cells through the collagen sponge, and especially through the spheroid, we took fluorescence microphotographs of the sponge with the spheroid around it showing green and red fluorescence, which indicates the successful passage of capsules regardless of size. Such a method was developed for the least losses during extraction of the spheroid from the sponge. After extraction, the spheroid was transferred to an empty plate, where it was also examined using confocal microscopy. By creating an orthogonal projection, we can clearly see the distribution of capsules inside the spheroid (Figure 4e,f). Capsules of 0.3 µm in size entered the inside of the spheroid in large numbers, which can clearly be seen in Figure 4d. Larger diameter capsules enter the spheroid to a much lesser extent. For example, 0.5 µm capsules are also present both on the upper cell layers and inside the spheroid itself, which can be clearly seen in Figure 4e. Particles of 4 µm are the least internalized. Only single capsules are observed in the center of the spheroid. There is a large amount of literature data, which indicates that the intercellular distance in the spheroid can reach from 20 to 500 µm [47]. However, the objects falling into the intercellular space can be larger, which depends on such parameters as the density of the intercellular matrix, and as in the case we describe the presence of the medium flow around the spheroid [48]. Our assumptions are also confirmed by the fluorescence profiles taken from cell spheroids with internalized capsules (Figure 4g). As can be seen from the graph, the highest signal was obtained from the spheroid, which was in the flow of 0.3 µm capsules. Further, depending on the size, the number of particles decreased significantly. To obtain quantitative data, we calculated capsule penetration depths for all three sizes (Figure 4h). The data were presented as frequency counts and Gaussians for these frequencies. Such a calculation allows us to determine the largest clusters of particles as a function of penetration depth. As can be seen in Figure 4h, most 4 µm particles accumulate mainly at the surface of the spheroid, 0.5 µm particles accumulate at a depth of ≈75 µm, as also seen from the Gaussian. Particles of 0.3 µm size penetrate most deeply into the cellular spheroid (≈125–150 µm from the surface).

## 3. Materials and Methods

### 3.1. Materials

Calcium chloride dihydrate, sodium carbonate (anhydrous), bovine serum albumin (BSA, lyophilized powder), tannic acid (TA), phosphate buffer solution (PBS), ethylene diamine tetraacetic acid disodium salt (EDTA), cardiogreen (ICG), hydrochloric acid, fetal bovine serum (FBS), RPMI-1640 medium, DMEM medium, pancreatin from porcine pancreas (≥100 USP U/mg), sodium chloride were purchased from Sigma-Aldrich (St. Louis, MO, USA). Penicillin, trypsin–EDTA, DPBS and streptomycin were purchased from Gibco, Spain. 2 wt.% of collagen in 0.02 M acetic acid solution was purchased from BioProduct Ltd., Moscow, Russia. All chemicals were used as received without further purification. All solutions for capsule preparation and biological experiments were prepared using deionized (DI) water (specific resistivity higher than 18.2 MΩ cm, Milli-Q plus 185 (Millipore, Burlington, MA, USA) water purification system). Polydimethylsiloxane elastomer was purchased from Dow Chemical, Midland, MI, USA (Sylgard 184).

### 3.2. Preparation of CaCO_3_ Particles

Submicron sized CaCO_3_ templates (400–600 nm in average diameter) were synthesized as described elsewhere [49]. In brief, 4 g of glycerol were mixed with 0.4 mL CaCl_2_ and 0.4 mL Na_2_CO_3_ aqueous solutions of equal concentration (0.5 M) under vigorous stirring at 700 rpm at room temperature. After 60 min, the solution became cloudy, indicating precipitation of CaCO_3_. After the next 60 min of continuous stirring, the suspension was centrifuged and the precipitate was washed with excess DI water five times to remove glycerol.

Submicron particles of vaterite with a size of about 300 nm were synthesized according to the protocol. Particles were prepared by mixing a solution of 10 mL 5 mM CaCl_2_ (in 85% ethylene glycol) and 10 mL 25 mM NaHCO_3_ (in 85% ethylene glycol) at 700 rpm at room temperature. After 15 min, the solution turned cloudy, which indicates the precipitation of CaCO_3_. Then the suspension was centrifuged, and the precipitate was washed several times with ethyl alcohol to remove ethylene glycol and dried.

Spherical porous CaCO_3_ particles with an average diameter of ~4 μm were synthesized according to Volodkin et al. [50]. In post-loading, 0.6 mL of 1M CaCl_2_ and Na_2_CO_3_ solutions were injected into 1.8 mL of DI water under vigorous agitation. 2 min later agitation was stopped and CaCO_3_ particles were separated by centrifugation and washed two times with DI water.

### 3.3. Capsules Preparation

The BSA-Cy5 loading of calcium carbonate cores was conducted with the novel freezing-induced loading (FIL) technique [51] using mini-rotator TetraQuant R-1 (TetraQuant LLC, Moscow, Russia). In short, 1 mL of CaCO_3_ particles sample was mixed with 1 mL of BSA-Cy5 solution. The obtained mixture was frozen under constant mixing at −20 °C for 2 h. After that, the sample was thawed and centrifuged at 8000 rpm for 2 min and washed 3 times with DI water. Capsule shell consisted of 2 bi-layer of BSA and TA (BSA-TA). After that, vaterite cores were dissolved with EDTA solution (0.2 M) in case of Parg/Dex capsules or 0.1 M HCl (in case of BSA/TA capsules), resulting in polymeric capsules being created. After each adsorption step, as well as after the dissolution of the cores, the suspension of capsules was purified by centrifugation (1 min, 8000 rpm) and double washed with DI water (2 mL).

### 3.4. Capsules Characterisation

Capsule concentration was measured with NTA (Nanoparticle Tracking Analysis) NanoSight (Malvern Ins. Ltd., Worcestershire, UK)

Scanning Electron Microscopy (SEM) analysis was performed using the scanning electron microscope (Helios G4 Plasma FIB Uxe). Samples were prepared by depositing a drop of a capsule suspension on a silicon wafer and allowing it to dry at room temperature. Before imaging, dried specimens were sputter-coated with approximately 5 nm thick gold film using a Denton sputter-coater.

Zetasizer Nano ZS (Malvern Ins. Ltd., Worcestershire, UK) was used to measure a zeta potential and a size of capsules. The obtained value of a size and a zeta potential was averaged three times.

### 3.5. Microfluidic Chip Fabrication

The presented organ-on-chip model was produced from silicone elastomer Sylgard 184, with the channel covered by glass. Silicone tubes are fitted to the chip body through stainless steel tubes.

The master mold for casting the chip body was created by photopolymer 3D printing using water washable resin Elegoo (Elegoo Inc., Shenzhen, China). When casting silicone elastomer, a separating layer was used to prevent the inhibition of the elastomer polymerization.

### 3.6. Formation of the Collagen Sponge—Model of Lymphoid Tissue

An open-porous sponge was formed from a collagen by the freeze-casting method [42,43]. A mold consisting of an aluminum substrate and 3D printed walls from polylactide (PLA) was used to form a porous material. 2 wt. % collagen gel in 0.02 M acetic acid was placed in the mold, where the directional crystallization was provided by the cooling of metal substrate with liquid nitrogen. Due to the thermal gradient, ice crystals grow perpendicular to the cooled mold wall and form a lamellar structure, pressing collagen molecules between ice lamellas. The pore size in the sponge depends on the freezing front speed and can be adjusted by changing the temperature of the cooled mold wall. We used liquid nitrogen to cool the sample, because it allow us to achieve high freezing front speed and get a sponge with a pore size close to one in a native collagen extracellular net [52]. After initial crystallization of the sample, it was freeze-dried in Free Zone bench-top device Labconco^®^ (Kansas City, MO, USA) at −30 °C for 48 h. After freeze-drying the collagen sponge has a structure with the open porosity. Then, the collagen sponge was stabilized using a carbodiimide crosslinking with EDC/NHS (Sigma-Aldrich, Burlington, MA, USA). A solution of 50 mM N-(3-dimethylaminopropyl)-N’-ethylcarbodiimide hydrochloride (EDC) and 25 mM N-hydroxysuccinimide (NHS) in 98% ethanol was used. Covalent crosslinking using EDC/NHS is a conventional method for treatment of collagen based materials [53,54]. The lyophilized collagen sponge was placed in EDC/NHS solution for 4 h, then washed in a PBS buffer for 1 day. The obtained porous material was sliced on thin pieces for the lymph node-on-chip (Figure 1e). To obtain piece of sponge with long interconnected pores we cut pieces of sponge from the area of the initial sample corresponding to steady-state growth zone during freeze-casting.

### 3.7. Cell Cultivation

4T1 and Jurkat cells were cultured in DMEM (Thermofisher, cat. # 10564011, Waltham, MA, USA) and RPMI 1640 (Thermofisher, cat. # 11875093) supplemented with 10% FBS, and 100 μg/mL penicillin/streptomycin, respectively. The media were replaced every 3 days, and the cells were maintained in a humidified incubator at 5% CO_2_ and 37 °C (Innova CO-170, New Brunswick Scientific, Edison, NJ, USA).

### 3.8. Formation of Tissue Spheroids

Tissue spheroids were formed from 4T1 cells using ultra-low adhesion Corning spheroid microplates (Corning, cat. # 4520) according to the manufacturer protocol. Briefly, monolayer cells with 95% confluence were rinsed by EDTA solution, harvested from the culture flasks by 0.25% trypsin/0.53 mM EDTA and then suspended in cell culture medium. The concentration of the cells was 1 × 10^6^ cell/mL. 10 μL of cell suspensions were dispensed to the wells of Corning spheroid microplates with 90 µL of culture medium. Corning spheroid microplates were incubated at 37 °C in a humidified atmosphere with 5% CO_2_ for 3 days.

### 3.9. Fluorescence Microscopy

For cell visualization we used fluorescence microscopy. Fluorescent images were obtained by Operetta CLS (Perkin Elmer, Waltham, MA, USA) microscope with 2×, 20× and 60× objectives and appropriate filters. Jurkat cells were stained with Calcein Am (ThermoFisher Scientific, Cat. # C1430). For this purpose, 0.5 µg of dye was added to 2 mL of cell medium containing 2 × 10^6^ cells. The cells were incubated for 15 min in the thermostat. After that, the cells were centrifuged at 700 rpm for 5 min. The supernatant was withdrawn and a new medium was added to the cells. The 4T1 spheroids were stained with Hoechst 33,342 (ThermoFisher Scientific, Cat. # H3570) at a concentration of 0.02 µg/mL for 15 min in the incubator. The spheroid was then washed in a PBS solution and transferred to a new medium. To obtain z-stacks, 30 photographs of the spheroid were taken along the z axis with a height difference of 10 µm. The experiments were evaluated using three fluorescence images spaced 75 µm apart in the spheroid. In each layer, we evaluated 20 random capsules. The penetration depth and fluorescence profile were analyzed using Imagej software.

## 4. Conclusions

A microfluidic lymph node-on-a-chip was developed. It allows us to operate with: (1) Lymph flow with T-cells through LNOC model tissues; (2) 3D structure of secondary tumor; (3) 3D collagen structure of lymph node tissues; (4) penetration of cancer cells into healthy LN tissues; (5) permeability of the affected lymph node perfused with T-cells and BSA/TA capsules; (6) interaction of different size capsules with 3D cell spheroid; and (7) permeability of model collagen tissues for capsules of different sizes, was developed. To demonstrate the applicability of the LNOC for pharmacological and diagnostic applications, we used a LNOC to evaluate the effect of polymer capsules at submicron and micron sizes on their internalization into metastasizing cells. For this purpose, lymphoid tissue was simulated in the chip by obtaining a collagen sponge of suitable porosity. To simulate secondary tumor in the LN, we obtained the spheroids based on 4T1 breast cancer cells, which fully reflect the interaction of cells in contrast to 2D cultures. To mimic the conditions of injection, the capsules were injected mixed with lymphocytes (basic elements of lymph) in a flow rate that matched that of the lymph flow. According to the obtained data, the possibility of delivering contrast agents increased with a decrease in the capsule size. Thus, in our study, 0.3 μm capsules, which penetrated well into the spheroid, turned out to be the most effective for delivery. These results highlight the potential of our LNOC for use in particle and contrast agent testing. They also demonstrate that the LNOC is an attractive alternative for further analysis and understanding of the effectiveness of developed drug delivery vehicles and contrast agents delivered to cells and organs. Moreover, some findings that we have obtained during the development of our device can be used for the elaboration of other types of lab-on-chip systems.

## Data Availability

All data presented here are adopted from the published work cited in the references.

## Supplementary Materials

The following supporting information can be downloaded at: https://www.mdpi.com/article/10.3390/ijms24043183/s1.
