# Supplementary material for: Plug-and-Play Lymph Node-on-Chip: Secondary Tumor Modeling by the Combination of Cell Spheroid, Collagen Sponge and T-Cells
Source: Int J Mol Sci. 2023 Feb 6;24(4):3183. doi: 10.3390/ijms24043183 (PMC9966643; doi:10.3390/ijms24043183)
Supplement: Supplementary file 1 [file ijms-24-03183-s001.zip › ijms-2077555-SM.pdf]

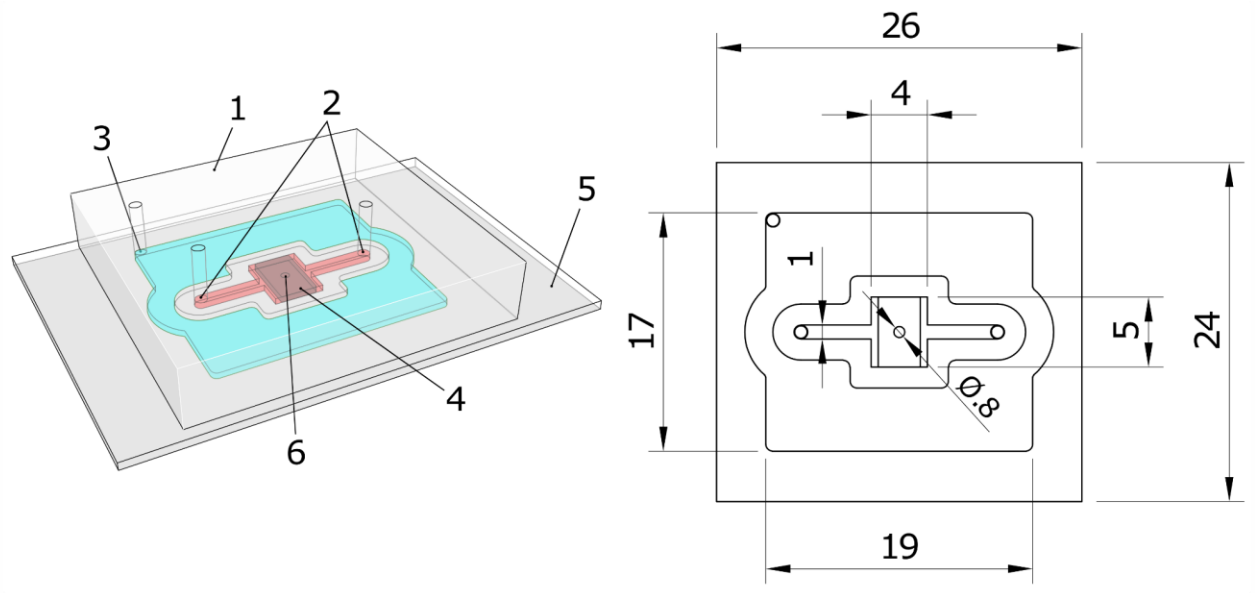

Figure S1. Schematic of the LNOC. 1 - chip body; 2 - main chanel with inlet and outlet; 3 - outlet of the suction cup; 4 - collagen sponge; 5 - glass for microscopy; 6 - hole in the collagen sponge for spheroid. Chanel height is 0.5 mm.

Video S1. Assembly and Application Process of the Lymph Node-on-Chip System
